# Residential Segregation of European and Non-European Migrants in Sweden: 1990–2012

**DOI:** 10.1007/s10680-018-9478-0

**Published:** 2018-03-21

**Authors:** Bo Malmberg, Eva K. Andersson, Michael M. Nielsen, Karen Haandrikman

**Affiliations:** Department of Human Geography, Stockholm University, Svante Arrhenius väg 8, 106 91 Stockholm, Sweden

**Keywords:** Ethnic residential segregation, Foreign-born, Migration, Multi-scalar, EquiPop, Sweden

## Abstract

In this paper, we analyse how a migrant population that is both expanding and changing in composition has affected the composition of Swedish neighbourhoods at different scales. The analysis is based on Swedish geocoded individual-level register data for the years 1990, 1997, 2005, and 2012. This allows us to compute and analyse the demographic composition of neighbourhoods that range in size from encompassing the nearest 100 individuals to the nearest 409,600 individuals. First, the results confirm earlier findings that migrants, especially those from non-European countries, face high levels of segregation in Sweden. Second, large increases in the non-European populations in combination with high levels of segregation have increased the proportion of non-European migrants living in neighbourhoods that already have high proportions of non-European migrants. Third, in contrast to what has been the established image of segregation trends in Sweden, and in an apparent contrast to the finding that non-European migrants increasingly live in migrant-dense neighbourhoods, our results show that segregation, when defined as an uneven distribution of different populations across residential contexts, is not increasing. On the contrary, for both European migrants from 1990 and non-European migrants from 1997, there is a downward trend in unevenness as measured by the dissimilarity index at all scale levels. However, if segregation is measured as differences in the neighbourhood concentration of migrants, segregation has increased.

**Electronic supplementary material:**

The online version of this article (10.1007/s10680-018-9478-0) contains supplementary material, which is available to authorized users.

## Introduction

Since the 1980s, Sweden has experienced high and increasing levels of international migration, with, in recent years, about 100,000 immigrants entering Sweden every year. As a result, one-sixth of the Swedish population is currently foreign-born. In addition, an increasing proportion of the migrant population originates from non-European countries. In this context, the aim of this paper is to analyse how a migrant population that is both expanding and changing in composition has affected the population composition of Swedish neighbourhoods in different parts of the country and at different geographical scales.

Within this migration history of expansion and change, residential segregation patterns and neighbourhood compositions are expected to change over time. Theoretically, spatial assimilation, place stratification, and ethnic preference may be at play in the Swedish context. Clearly, patterns of spatial assimilation can have played an important role for migrants that have been in Sweden for a long time. Studies have shown that newly arrived migrants tend to be more vulnerable on the housing and labour market compared to natives, but with time, employment and situated knowledge make migrants’ housing careers become more similar to natives’ housing careers. On the other hand, mechanisms related to place stratification also seem to apply (Turner and Hedman 2014). Even if some migrants move out from immigrant-dense areas as they become well established in society, other groups of migrants become permanent residents of typical migrant neighbourhoods (Bråmå 2006). Some migrants face discrimination and become stuck in stigmatised areas, unable to make a housing career, as suggested by spatial assimilation theory. Others have argued that this pattern instead reflects ethnic preferences.

Also relevant for a study of how migration affects neighbourhood composition are studies of increasing ethnic diversity in high-income country contexts (Vertovec 2007). In the USA, this literature focuses mainly on racial diversity (Clark et al. 2015), whereas in Europe, ethnic and cultural diversity have been given more attention (Tasan-Kok et al. 2013). One important question has been if processes towards more residential mixing can be linked to a simultaneous process where ethnicity and race become less important perceptual categories and ethnic preference, thus, loses some of its importance as a determinant of residential choice and cause of segregation (Clark et al. 2018).

In the literature, segregation patterns have traditionally been identified using data aggregated for fixed geographical subdivisions, such as census tracts. However, as argued by Clark et al. (2015), using fixed geographical subdivisions has several drawbacks. First, there is the modifiable areal unit problem that makes measurement dependent on the way areal units are delineated. Second, with fixed geographical subdivisions it can be difficult to assess the geographical scale of segregation patterns (Lichter et al. 2015; Reardon et al. 2008). Third, a central concern in relation to segregation is the way individuals are affected by their geographical context, and in many cases the statistical area in which people live does not give a good representation of this context (Kwan 2012). Therefore, in this paper, the analysis of changing neighbourhood composition is based on the use of individualised, scalable neighbourhoods. The advantages of this approach compared to using fixed areas are demonstrated by Östh et al. (2014b). From a conceptual point of view, using individualised neighbourhoods represents a change away from a traditional Chicago-school approach that theorises neighbourhoods as distinct social units. With an individual perspective, the number of neighbourhoods is instead equal to the number of individuals, since every person has his or her own neighbourhood. As discussed in Hipp and Boessen (2013), acknowledging that individuals essentially have their own neighbourhood, or ego-hood, will have consequences for how neighbourhoods are theorised. There will be substantial variation in the size of the geographic areas of individualised neighbourhoods based on different rates of population density. Therefore, while the notion of the individualised neighbourhood, consisting of for example one’s closest 200 neighbours, holds true, its geographical manifestation will be very different in a highly populated city (a few buildings) compared to sparsely populated rural areas (possibly a radius of several kilometres). This needs to be taken into account when interpreting the results. The reverse interpretation problem would be found if one had equally sized geographical areas as a basis for study; the geographical size would be the same but the populations would vary across areal units.

One aspect of segregation that has been brought to the fore in studies using individualised neighbourhoods is the importance of scale. This will also be mirrored in the present study. Segregation can occur on multiple geographical scales and there is a need to be specific about on which geographical scale different theories of segregation are assumed to be operative. We acknowledge that progress towards a more nuanced understanding of the scalar aspects of segregation requires an interplay between empirical studies and conceptual development, but in the present study the emphasis will be on identifying empirical patterns.

## Background: Changing Ethnic Residential Segregation Patterns in Sweden

Immigration to Sweden that is of interest for this study dates back to the 1950s and 1960s. In the 1950s, migrants came mainly from Europe, mostly from Finland, Italy, the former Yugoslavia, and Greece (Nilsson 2004). This mainly traditional labour migration meant that migrants started working in the Swedish manufacturing industry immediately upon arrival. The residential patterns of these migrants reflected the industry’s need for labour: medium-sized cities with manufacturing industry received the most immigrants. Later migrants were often refugees, following the military coup in Chile (1972), wars in Iran, the former Yugoslavia (1990s), and Somalia, and they faced longer waiting times for residential permits and before being allowed to enter the labour market (Grand and Szulkin 2002). Their residential patterns were also subject to governmental dispersal policies such as the ‘whole of Sweden strategy’ in the late 1980s (Åslund 2005). Today, asylum seekers, mostly from Iran, Syria, and other Middle Eastern countries, make up the largest group of immigrants to Sweden. Since 1994, new legislation has given asylum seekers in Sweden a right to settle more freely (Myrberg 2017).

Ethnic residential segregation levels in the metropolitan areas of Sweden have been characterised as rather low compared to levels found in North America (Marcińczak et al. 2016; Murdie and Borgegård 1998), and perhaps with levels more comparable to Australian and Canadian metropolitan areas (Murdie and Borgegård 1998). Migrants from the global south have the highest levels of residential segregation from Swedish-born residents, but in general there is a growing neighbourhood mix, with a decrease in native neighbourhoods and a concurrent increase in mixed neighbourhoods (Marcińczak et al. 2016).

Most longitudinal studies of ethnic residential segregation in Sweden focus on metropolitan areas. These studies are of different types. First, there is a group of studies that measures segregation trends in a single metropolitan area using administratively defined areas such as SAMS areas or parishes, and the dissimilarity index or the index of segregation (Andersson et al. 2006, 2007, 2009; Andersson and Kährik 2016; Marcińczak et al. 2016; Murdie and Borgegård 1998). Second, there are studies on segregation trends that focus both on residential segregation and school segregation (Böhlmark et al. 2015; Holmlund et al. 2014; Lindbom 2010; Lindbom and Almgren 2007; Nordström Skans and Åslund 2010). Third, there are studies published by the Swedish National Board of Health and Welfare that use the entropy index to study trends in ethnic segregation (Biterman 2006, 2010; Biterman and Franzén 2007). Fourth, a few media organisations have initiated their own studies on the question of whether segregation is increasing (Dagens Nyheter 2015; Sveriges Radio 2012). Finally, recent studies have been published using individualised neighbourhoods to analyse segregation trends in Stockholm county and Skåne county (Niedomysl et al. 2015; Östh et al. 2014a).

The results of these studies are difficult to compare since the studies use different geographical units and different measures of segregation, compare different time periods, and use different definitions of migrant groups. In general, however, these studies conclude that segregation has increased over time. For instance, Niedomysl et al. (2015: 4) conclude that in Skåne, “residential segregation of the foreign-born has increased over time”. Östh et al. (2014a) draw the same conclusion and add “visible minorities are more and more concentrated in places that already have a high concentration of visible minorities”. In 2012, the news service of the Swedish national radio reported, based on a commissioned study, that “residential segregation has doubled during the last 20 years” (Sveriges Radio 2012). Similarly, Sweden’s largest newspaper examined ethnic segregation in the 30 largest municipalities in Sweden for the period 1991–2013 and concluded “ethnic segregation has increased in all these municipalities except two” (Dagens Nyheter 2015). Biterman’s (2010) study of the three major metropolitan areas for the period 1990–2006 states that “segregation has increased in all three regions”. Nordström Skans and Åslund (2010: 91), after pointing to high levels of segregation, are somewhat more cautious, stating “segregation seems to have increased over time, too”.

In contrast, in their report on Malmö, Andersson et al. (2007) do not claim that ethnic segregation increased during the period 1990–2004, but discuss mechanisms behind segregation in a way that could suggest to the reader that segregation is increasing: “The report focuses on the behaviour of the majority population since their behaviour has decisive importance for ethnic residential segregation, partly because the majority leaves certain areas that gain a larger minority population, partly because the majority population avoids moving to minority dominated areas” (p. 6). Nevertheless, they present, but do not discuss, figures showing declining values for the dissimilarity index for most immigrant groups between 1995 and 2004. A similar analysis of the Gothenburg region also reports declining dissimilarity index for most immigrant groups, but this is not reflected in the conclusions (Andersson et al. 2009).

Earlier, Murdie and Borgegård (1998) examined ethnic segregation among 12 immigrant groups in Stockholm parishes using the dissimilarity index, and reported increased levels in the 1990s among some newly arrived groups, such as Ethiopians, Iraqis, and Somalis, while segregation levels among other groups remained stable or even declined. A recent study on migrant segregation based on SAMS areas in Stockholm found increasing segregation levels for both native Swedes and migrants from former socialist countries, but decreasing levels for migrants from the global south and Western countries, based on the index of segregation (Marcińczak et al. 2016).

Thus, in many Swedish studies the emphasis has been on increasing ethnic segregation, though their results based on the dissimilarity index often showed declining segregation. This is in contrast to a number of international studies that have called attention to declining levels of segregation based on trends in the dissimilarity index (Frey 2014; Shon and Verdugo 2015; Simpson 2007). Since assessing segregation trends is such an important issue, both academically and politically, a critical perspective on the methods used in such studies is warranted. Thus, this paper will discuss in what terms changing segregation patterns can be described.

Based on earlier studies and the theories of spatial assimilation and place stratification, we formulate the following hypotheses for the two decades under study:

1. The concentration of non-European migrants will increase both in areas with low and high concentrations of non-European migrants. This is a consequence of the expansion of the non-European population and will be observed at all scale levels.
2. Since the European migrant population has been relatively stable over time, there will be little change in the neighbourhood proportion of this population across most scale levels.
3. Since the European migrant population has had a longer presence in Sweden than the non-European population, we expect the dissimilarity index for this group to have declined as a consequence of spatial assimilation. We expect this trend to be visible across different scale levels.
4. The trend in the dissimilarity index for the non-European population is more uncertain. Given the results of earlier studies, we expect that it has not increased but it is possible that it has in fact declined. There is a possibility that large-scale segregation has increased for the non-European population.
5. Earlier studies report that the dissimilarity index is higher for non-European than for European migrants. An increasing weight of non-Europeans in the migrant population may have the effect of increasing the dissimilarity index for the total migrant population, and this is possible across all scale levels.

## Study Design: Data and Method

Our analysis of changing neighbourhood composition focuses on three different aggregations of the foreign-born population. We used a combination of descriptive tools in the analysis: percentile plots, aggregate segregation indices and maps based on a multi-scalar classification of neighbourhood types.

### Migrants in Sweden: Definition and Data

How ethnic residential segregation is measured depends to a large extent on definitions by statistical offices on race, ethnicity, and migrant background. In Sweden, guidelines recommend using the country of birth of a person and his or her parents, or alternatively citizenship, as defining characteristics (Statistics Sweden 2002). The term ‘race’ is very rarely used in contemporary Swedish society and research, although the idea of Swedishness based on historical and cultural claims still exists (Osanami Törngren 2011). Ethnicity indicates some kind of shared culture and is often ascribed to people who look non-Western, with the majority population in Western countries not usually identifying themselves as “ethnic” (Frankenberg 1993). According to Mattsson (2005), Swedishness is strongly connected to visible European whiteness. In research on segregation concerning immigrants, the concept of ethnicity is often used and we will sometimes adhere to that term as well. However, ethnicity is related to belonging to a group formed by origin or culture that in some way can be seen as distinct from other groups. In this sense, origin might be kinship, history, or geographical origin. Assuming ethnicity from country of birth is thus not possible.

This study defines a migrant as a person born outside Sweden, excluding those with one or more parents born in Sweden. The migrant population is subdivided into two groups: European migrants and non-European migrants. European migrants include all migrants born in Europe, including Turkey. Non-European migrants are individuals born in Africa, South America, or Asia. Migrants from North America, Australia, and New Zealand have been excluded because of the small size of these migrant groups (below 5% of the migrant population in all years). The exclusion of these migrant groups implies that the non-European migrant group consists of individuals that in the Swedish context form a visible minority who may experience housing and labour market discrimination (Molina 1997).

The analyses in this paper are based on register data collected in the so-called GEOSTAR database, managed by the Department of Human Geography at Stockholm University. The data are accessed through Statistics Sweden’ system of Micro-Online Access (MONA). GEOSTAR includes all individuals in the Swedish population during the period 1990–2012 and includes a wide range of demographic, socioeconomic, and geographic variables. All individuals were assigned coordinates based on primary residence, resulting in a geocoded database for the whole population. The coordinates were provided by Statistics Sweden, aggregated based on the classification of urban areas (*tätortsindelning*). The classification of urban areas is updated every 5 years, with urban areas being delineated as contiguous builtup areas with a maximum of 200 metres between buildings and including at least 200 residents. Some special rules apply to farm buildings, parking lots, and so on (Statistics Sweden 2011). Due to privacy concerns, Statistics Sweden aggregated the coordinates delivered to GEOSTAR to 250 m squares in urban areas and 1000 m squares in rural areas, based on the 2010 urban classification. The number of 250 m squares increased from 90,922 in 1990 to 96,043 in 2012, whereas the number of 1000 m squares decreased from 111,185 in 1990 to 106,047 in 2012. For a detailed description of the data see Nielsen et al. (2017).

**Fig. 1:**
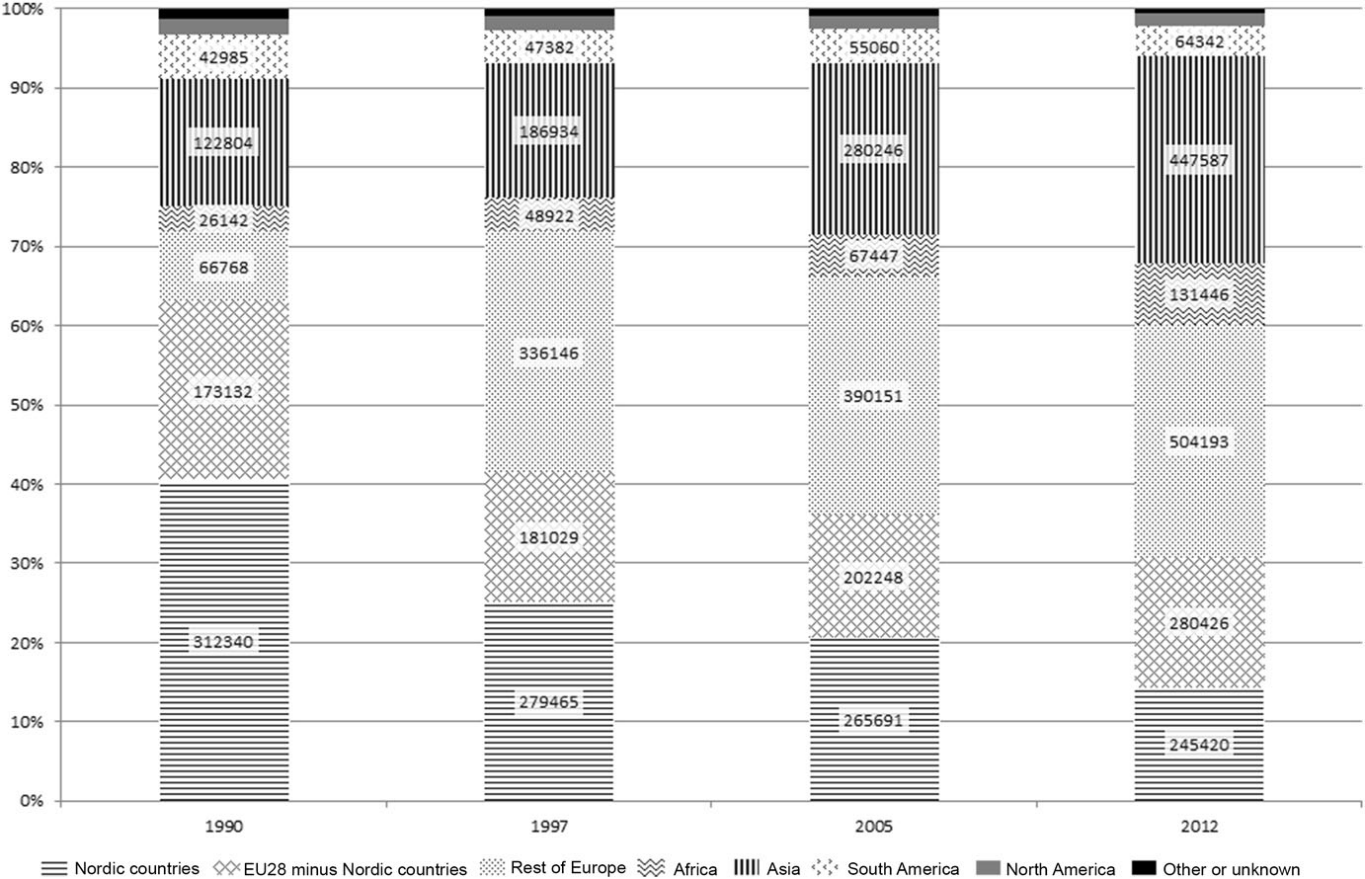
Migrant population in Sweden by origin, 1990–2012. *Source data*: Swedish register data, authors’ calculations

Figure 1 shows the changing composition of the Swedish migrant population for the years included in the study, by region of birth categories available in GEOSTAR. The figure shows that overall, the proportion of European migrants among the total migrant population has decreased. While in 1990, European migrants represented almost three-quarters of all migrants, in 2012 they made up 60 percent of the migrant population, as a consequence of the growth of the non-European migrant population and a decrease in migrants from other Nordic countries, notably Finland, and a decrease in EU28 migrants. This trend has not been changed by the fact that the proportion of migrants from South-Eastern Europe increased over time, especially from the former Yugoslavia from the second half of the 1990s, and from Poland from the 2010s. Most non-Europeans originate from Asia; this proportion increased from 16 per cent of all migrants in 1990 to 26 per cent in 2012. Among Asians in Sweden, Iraqis form the largest group, followed by Iranians and Syrians. Summarising, Sweden’s migrant population is increasingly characterised by diversity of country of birth.

### Individualised Neighbourhoods

Our analysis of changing neighbourhood composition was based on the use of individualised, or what is sometimes called ‘egocentric’, scalable neighbourhoods. To calculate the population composition of an individualised neighbourhood using geocoded data we proceeded in three steps. First, we expanded a circular buffer around the centres of populated grids cells until the population contained in the buffer reached the specified threshold. Second, for the grid cells contained in the buffer, we examined the total number of individuals belonging to the migrant group under study. Third, the proportion of migrants in the buffer population was computed. This procedure identified the *k*-nearest neighbours of different individuals, where *k* is the size of the threshold population. Five different threshold populations, or neighbourhood scale levels, were used (*k*): the 100, 800, 6400, 51,200, and 409,600 nearest neighbours. Values obtained for large *k* correspond to large-scale neighbourhoods. Values obtained for small *k* correspond to small-scale neighbourhoods. To construct buffers and compute the population composition of individualised neighbourhoods we used the EquiPop software (Östh 2014; Östh et al. 2014b). The proportion of all migrants, European migrants, and non-European migrants in individualised neighbourhoods were calculated for the years 1990, 1997, 2005, and 2012, and for the five different scale levels mentioned above.

As noted earlier, the physical size of neighbourhoods varies with population density; in locations with increasing population density, the radius corresponding to a specific population threshold will decrease over time. In principle, this implies that the population of an individualised neighbourhood could become more homogenous as a result of local population growth. For example, a neighbourhood that formerly included both single family and multifamily housing could change into a neighbourhood that only contains multifamily housing if an increasing population leads to a decline in the radius required to reach the threshold population. In practice, this is not likely to influence our results to a large extent, since larger shifts in the radius are a rare occurrence.

### Percentile Plots

To analyse the variation in population composition across individualised neighbourhoods, we used percentile plots. When individualised neighbourhoods are used, the number of neighbourhoods corresponds to the number of individuals in the study population. For example, in 1990 the resident population in Sweden was 8,590,701; thus, there were 8,590,701 individualised neighbourhoods. In the last year covered by our study, 2012, the Swedish population had expanded to 9,555,893. Percentile plots give a comprehensive picture of differences in neighbourhood composition by showing the proportion of neighbourhoods above or below certain values for the migrant proportion in the population. By comparing percentile plots for different years, changes in the neighbourhood composition can be analysed. In addition to using all neighbourhoods, we also studied the neighbourhoods of three sub-populations: European migrants, non-European migrants, and the total migrant population. This allowed for an analysis of variation in neighbourhood composition from the perspective of all Swedish residents, European migrants, non-European migrants, and all migrants.

### Dissimilarity Index

To measure levels of segregation, aggregate indices were calculated using the dissimilarity index (see Duncan and Duncan 1955). The indices were constructed to be applied to situations with fixed geographical units but can be used for individualised neighbourhoods by treating each person’s individualised neighbourhood as equivalent to a fixed unit.

The dissimilarity index measures the evenness of the distribution of two groups across neighbourhoods (Massey and Denton 1988). In contrast to the isolation index, it is not sensitive to the size of the migrant population. The dissimilarity index is calculated using the formula:

$$D = \frac{1}{2}\sum\limits_{i = 1}^{S} {\left| {\frac{{m_{i} }}{M} - \frac{{n_{i} }}{N}} \right|}$$

where *i* is an individual; *S* is the whole population studied; *m*_*i*_ is the proportion of migrants in the individualised neighbourhood of *i; M* is the sum of all *m*_*i*_; *n*_i_ is the proportion of non-migrants in the individualised neighbourhood of *i;* and *N* is the sum of all *n*_*i*_. Note that in this formula, *M*, *m*_*i*_, *N*, and *n*_*i*_ are not defined in the same way as when DI is computed for fixed geographical areas. With this definition, the dissimilarity index based on individualised neighbourhoods has the same properties as when the dissimilarity is computed on data for fixed geographical areas, including the property that it shows the proportion of the minority population that needs to move in order to arrive at an even distribution. This can be seen from the above formula where the absolute difference between the neighbourhood share of the aggregate minority and aggregate non-minority population shows the adjustment that needs to be made to the minority population in order to have an equal representation of migrants and non-migrants across different neighbourhoods.^1^

### Standard Deviation in the Neighbourhood Proportion of Migrants

In addition to the dissimilarity index, we also computed the standard deviation in the neighbourhood concentration of migrants as an alternative aggregate measure of segregation. In contrast to the dissimilarity index, this standard deviation is sensitive to the overall size of the migrant group. Thus, it provides a complementary measure of differences in the proportion of migrants between neighbourhoods, and it can be argued that it captures an important aspect of segregation: segregation as variation in geographical context (see Jones et al. 2015).

### Factor and Cluster Analysis to Reveal Geographical Patterns

Although the percentile plots are useful for analysing changes in neighbourhood composition, they do not provide information about the geographical distribution of different neighbourhood types. Given the richness of the data on neighbourhood composition (four years, three migrant groups, five different scale levels and more than 200,000 different locations), analysing spatial patterns is challenging. We used cluster analysis to classify all locations into distinct types based on the composition of the neighbourhood population in the four years under study. A cluster analysis could have been applied to the complete data (60 different variables for each location) but given the high correlation between the variables, especially between different scale levels, we used factor analysis to capture the most important dimensions in our data, and subsequently used factor scores on these dimensions in the cluster analysis. There is a risk that a cluster analysis based on factor scores may fail to build interesting groups. But in our case, the aim was to capture general spatial patterns and, therefore, basing the classification on factor scores was helpful in establishing a relatively easy-to-interpret classification.

The factor analysis of the neighbourhood data suggested that three factors accounted for most of the variation. The loadings for these factors are presented in the table shown in Fig. 5. Two of these factors represent the presence of European migrants (factor 1) and non-European migrants (factor 3), respectively. Factor 1 and factor 3 have the highest loadings for scales that correspond to the size of local neighbourhoods, *k *= 6400 and *k *= 800. Factor 2, on the other hand, has low loadings for scale levels that correspond to the local neighbourhood (*k *= 100, *k *= 800, *k *= 6400) and high loadings for scale levels that correspond to the size of urban areas (*k *= 51,200 and *k *= 409,600).

The classification of individualised neighbourhoods into different types was done with hierarchical cluster analysis using Ward’s method. Initially, 20 different clusters were considered, but 12 clusters sufficed to capture the most important patterns. The more parsimonious approach was preferred in order to make the classification easier to grasp. The resulting clusters fall into three categories. First, a class of clusters is characterised by rising proportions of non-European migrants at the scale of the local neighbourhood but not necessarily at the scale of urban areas. Second, another class of clusters is characterised by low proportions of non-European migrants at the scale of the local neighbourhood and high proportions of non-European migrants at the scale of urban areas. A third class of clusters that was distinguished describes low proportions of non-European migrants both at the scale of the local neighbourhood and at the scale of urban areas. A more detailed account of the clusters is provided in Sect. 4.5.

## Results

**Fig. 2:**
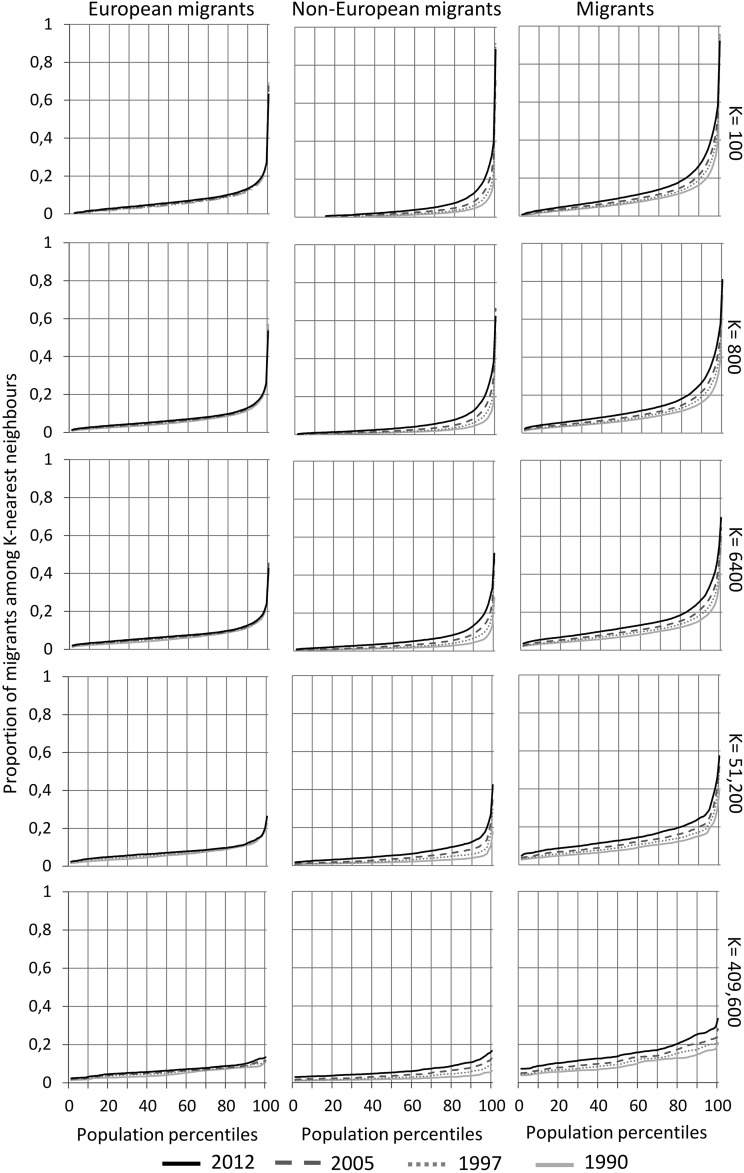
Individualised neighbourhoods’ proportions of European, non-European and total migrant (columns), for five scale levels (rows) and four years. Percentiles based on the total Swedish population. *Source data*: Swedish register data, authors’ calculations

**Fig. 3:**
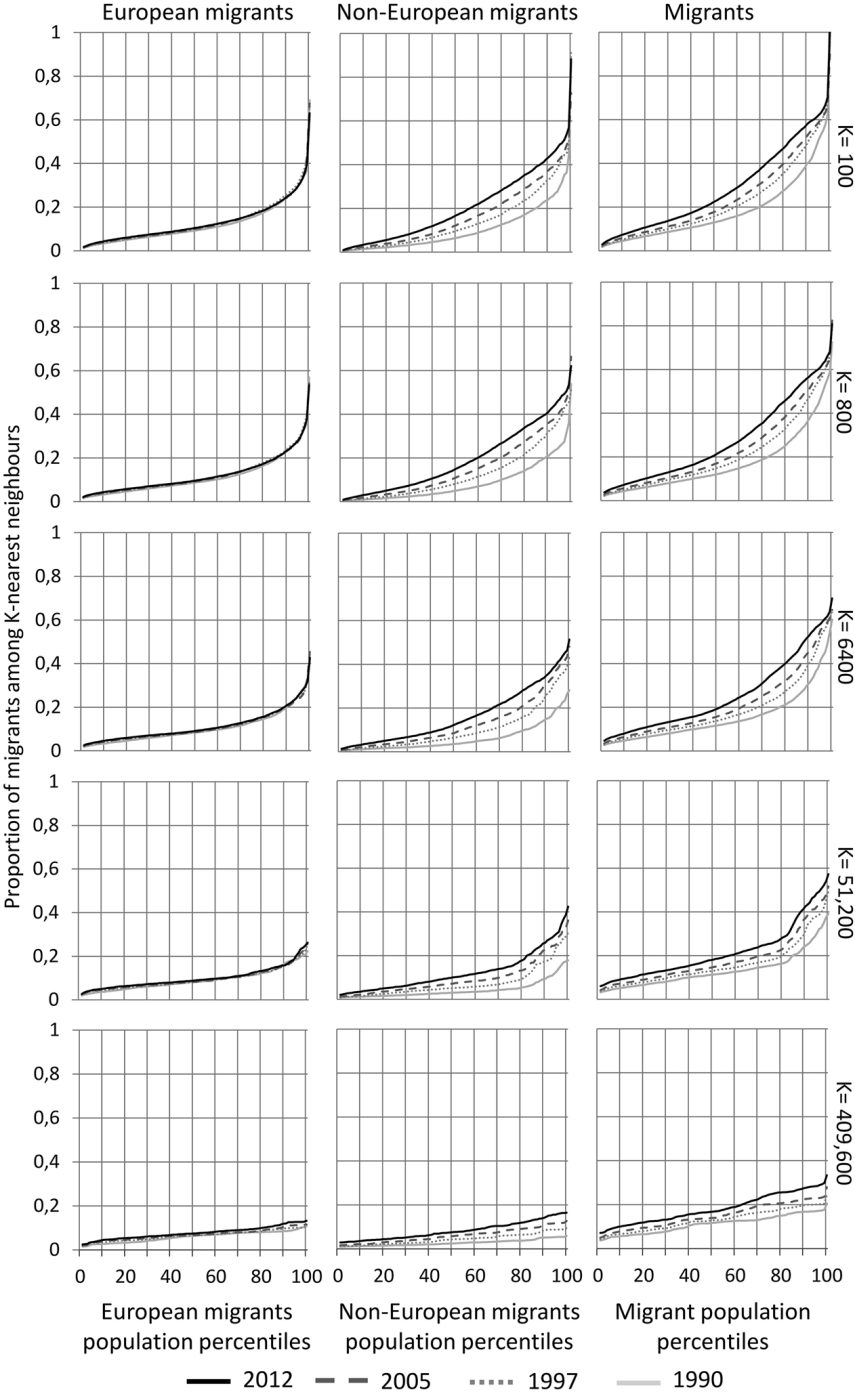
Individualised neighbourhoods’ proportions of European, non-European and total migrant (columns), for five scale levels (rows) and four years. Percentiles based on the European, non-European and total migrant populations. *Source data*: Swedish register data, authors’ calculations

The percentile plots are presented in Figs. 2 and 3. Each figure contains 15 different graphs, one for every combination of migrant group and neighbourhood scale level. The lines represent the variation in neighbourhood context for the years under study. Figure 2 presents the results with percentiles based on the entire Swedish population, while Fig. 3 presents the results with percentiles based on the three sub-populations European migrants, non-European migrants, and the total migrant population. Below, we analyse the segregation patterns of all migrants, non-European migrants, and European migrants based on these figures.

### Variation in the Concentration of Migrants Across Individualised Neighbourhoods

The percentile values for the proportion of the entire migrant population are in the right-most column of Fig. 2. Reading from the top to the bottom of Fig. 2 it is evident that, across all neighbourhood sizes and across all percentiles, there has been an increase in the proportion of migrants. In terms of percentage points, the increase is larger for higher percentiles compared to lower percentiles, but in relative terms the increase is at least as strong for the lower percentiles. The proportion of migrants in the most immigrant-dense neighbourhoods is generally higher in smaller individualised neighbourhoods compared to larger neighbourhoods. This is to be expected as small neighbourhoods can more easily include a homogenous population while larger neighbourhoods, for example with *k *= 409,600, become more mixed due to their sheer size. Overall, Figs. 2 and 3 show that migrants living in Sweden in 2012 lived in neighbourhoods with much higher proportions of migrants than migrants living in Sweden in 1990. In the general debate, the increasing proportion of migrants living in migrant-dominated neighbourhoods is often seen as a reason for concern. A reason for this could be that unemployment and social allowance dependence is high among migrants and that concentrated poverty can affect people in a negative way. In addition, a concern about consequences such as reduced social cohesion in the society overall is widely discussed (Tasan-Kok et al. 2013; Van Kempen and Bolt 2009).

### Variation in the Concentration of Non-European Migrants Across Individualised Neighbourhoods

The percentile values for the proportion of non-European migrants in differently sized individualised neighbourhoods are shown in the middle column of Fig. 2. The percentile plots in Fig. 2 clearly reflect the strong increase in the non-European migrant population between 1990 and 2012, thereby confirming hypothesis 1, and show a highly segregated pattern overall. However, the increase is unevenly distributed. Across different scale levels and across all years, 50% of the Swedish population lived in neighbourhoods where the proportion of non-European migrants could be considered low. This was true also in the last year of observation despite a strong increase in the non-European population. Especially notable is the total absence of non-European migrants for all years at the *k*-100 level in the 10 percentage most native-dominated neighbourhoods. In 1990, over 30% of the Swedish population had no non-European migrants among their nearest 100 neighbours. The low proportion of non-European migrants in non-migrant-dominated neighbourhoods is in sharp contrast to the rapidly rising proportions of non-European migrants in the most migrant-dense neighbourhoods.

A comparison across years shows that for *k *= 100 and *k *= 800 neighbourhoods, the relative increase in non-European migrants in the lower percentiles between 1990 and 2012 was faster than the relative increase in non-Europeans in the highest percentiles. This means that over time, non-Europeans are to a larger extent moving into neighbourhoods dominated by a low proportion of migrants, possibly as a result of dispersal policies. Last but not least, the increase in non-Europeans in areas with low proportions of migrants was faster than the overall increase in non-European immigrants at all *k*-levels for the period 1997 to 2012. This can be interpreted as showing tendency for more mixing and less reinforcement of non-European residential segregation patterns over this period.

Even though there has been some increase in the proportion of non-Europeans living in areas with few non-European migrants, the slope of the percentile-plot curve shows an even larger increase in the most concentrated areas. Consider for example the *k *= 800 neighbourhood level in Fig. 2. In 1990, the proportion of non-European migrants in the 90th population percentile was 5%, implying that 90% of the population lived in areas with less than 5% non-European migrants among their 800 closest neighbours. In 2012, the 90th percentile value had increased to 18%, meaning that 10% of the population, those belonging to the 90th to 100th percentiles, lived in neighbourhoods in which 18% or more of their 800 closest neighbours were non-European migrants. The establishment of neighbourhoods with large concentrations of non-European migrants has led to an increasing interest in segregation in the Swedish debate.

The large changes in neighbourhood composition that are associated with increases in the non-European population are also clearly shown in the percentile plots of Fig. 3 (middle column) where the population composition for percentiles based on the non-European migrant population is presented. For example, in 1990 only about 5% of non-European migrants lived in areas with more than 20% non-Europeans among their closest 6400 neighbours. This could be interpreted to mean that in 1990, relatively large-scale neighbourhoods with a dense non-European migrant population were close to non-existent, and that living in such areas was not a common experience for non-European migrants. In contrast, in 2012, 34% of the non-European migrants lived in neighbourhoods with 20% or more non-Europeans among their closest 6400 neighbours. Thus, in 2012, an experience that in 1990 was the exception had become a relatively common experience for non-European migrants living in Sweden.

The presumption that high concentrations of non-European migrants imply negative contextual effects has the drawback of failing to acknowledge positive contextual effects of living with co-ethnics. Upon entering a new country, migrants’ daily life may be eased by having countrymen around with the same language who can help migrants in finding their way around (food, traditions, child care, social interaction) in a new society. Positive effects described as defence, support, preservation, and even attack are used in discussions on ethnic preferences. For a good discussion see Knox and Pinch (2010).

### Variation in the Concentration of European Migrants Across Individualised Neighbourhoods

Lastly, segregation patterns for European migrants, found in the left column in Fig. 2, show few differences between the years of measurement, thereby confirming hypothesis 2. This is because the increase in European migrants is relatively small and relatively evenly distributed over the different percentiles, compared to non-European migrants in the central column. For example, 3% of the population had no European migrants among their 100 nearest neighbours in 1990, compared to 32% living in areas with no non-European migrants. In 2012, only 1% percentage of the Swedish population had no European migrants among their 100 nearest neighbours, compared to 12% who had no non-European migrants for the same *k*-level. One possible explanation for this is that many European migrants entered Sweden in the 1960s and 1970s and thus had longer to establish themselves in the housing and labour markets (Turner and Hedman 2014).

Figure 2 shows the proportion of European migrants among the closest 800 neighbours in the left column, second row. Proportions of European migrants are not particularly high below the 90th population percentile—the highest proportion occurred in 2012. For example, the proportion of European migrants at the *k*-100 level is less than 6% for up to half of the total population.

The left column of Fig. 3 presents the neighbourhood proportions of European migrants for percentiles based on European migrants. Here one can see, for example, that in 1990 for *k *= 6400 neighbourhoods, 10% of European migrants lived in neighbourhoods with at least 20% European migrants. By 2012, however, the proportion of European migrants living in European migrant-dense neighbourhoods had not increased as much as the sixfold increase for the proportion of non-European migrants living in non-European migrant-dense neighbourhoods. This difference in the segregation experience of European and non-European migrants can be seen as reflecting the changing composition of the migrant population in Sweden.

### Has Segregation Increased in Sweden?

The overall trend in the percentile plots of Figs. 2 and 3 is an increase in the proportion of migrants and non-European migrants in particular, in a majority of neighbourhoods in the period 1990–2012. An important question is whether there has also been an increase in segregation. To answer this question, we have used the most commonly used measure of segregation in the literature, the dissimilarity index.

Looking at the trends in the dissimilarity index in Table 1, it is clear that there is very little evidence for increasing segregation. On the contrary, for Europeans (from 1990) and for non-Europeans (from 1997), segregation as measured by the dissimilarity index is declining, confirming hypotheses 3. The reason why the downward trends in segregation of European and non-European migrants do not translate into a similar clear downward trend in the segregation of all migrants is the shift in the composition of the migrant population from European to non-European migrants. Since the latter population is more segregated than the former, the increasing weight of the non-European population counteracts the tendency towards lower segregation when the two groups are considered separately. In other words, on some *k*-levels, migrants in general become more segregated over time, but this is because the proportion of non-European migrants, who live in a much more segregated manner, is increasing over time. The clear decline in the dissimilarity index for European migrants across all scales confirms hypothesis 3. The mixed pattern for the trends in the dissimilarity index for all migrants partly confirms hypothesis 5. Hypothesis 4 is mostly confirmed since the dissimilarity index for non-European migrants has declined since 1997. Contrary to our expectation, this decline also occurred for the largest *k*-levels.

**Table 1:** Dissimilarity index for European migrants, non-European migrants and all migrants for different *k*-levels and years. *Source data*: Swedish register data, authors’ calculations

Immigrant group	*k*-level	1990	1997	2005	2012	Trend
Europeans	100	0.35	0.35	0.32	0.31	Decline from 1997
	800	0.31	0.31	0.29	0.27	Decline from 1997
	6400	0.28	0.26	0.25	0.24	Decline from 1990
	51,200	0.24	0.21	0.20	0.19	Decline from 1990
	409,600	0.23	0.19	0.17	0.17	Decline from 1990
Non-Europeans	100	0.54	0.55	0.53	0.51	Decline from 1997
	800	0.46	0.50	0.49	0.46	Decline from 1997
	6400	0.36	0.42	0.41	0.39	Decline from 1997
	51,200	0.27	0.32	0.32	0.30	Decline from 2005
	409,600	0.20	0.25	0.25	0.22	Decline from 2005
All migrants	100	0.37	0.39	0.39	0.40	Increase
	800	0.33	0.36	0.36	0.37	Increase
	6400	0.29	0.30	0.31	0.32	Increase
	51,200	0.25	0.25	0.25	0.25	Stable over time
	409,600	0.20	0.20	0.20	0.19	Decline from 2005

**Fig. 4:**
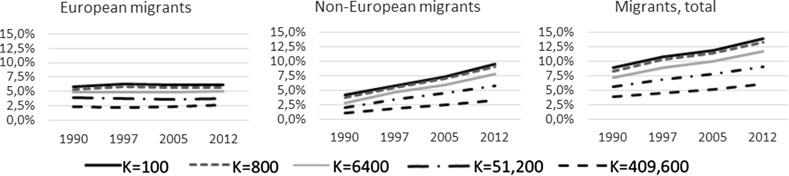
Standard deviation of migrant proportions in differently sized individualised neighbourhoods 1990–2012, European migrants, non-European migrants, and total migrants. *Source data*: Swedish register data, authors’ calculations

This finding of declining dissimilarity indices for both European and non-European migrants goes against popular beliefs that segregation is increasing. We would argue that one reason why trends in the dissimilarity index do not correspond to popular beliefs is that the dissimilarity indices fail to capture an important aspect of segregation: segregation as a variation in geographical context. This variation can be assessed by computing the standard deviation in the proportion of migrants across neighbourhoods. As shown in Fig. 4, there have been very large increases in the variation in neighbourhood composition both with respect to non-European migrants and with respect to the migrant population, and these increases correspond to an increasing slope of the corresponding percentile plots. This increased variation can be experienced as increased segregation, and the fact that this increased variance is not captured by the dissimilarity index points to a need not to rely solely on the dissimilarity index when segregation trends are assessed. For a more extensive discussion see Andersson et al. (2018).

### Geographical Patterns of Neighbourhood Change

**Fig. 5:**
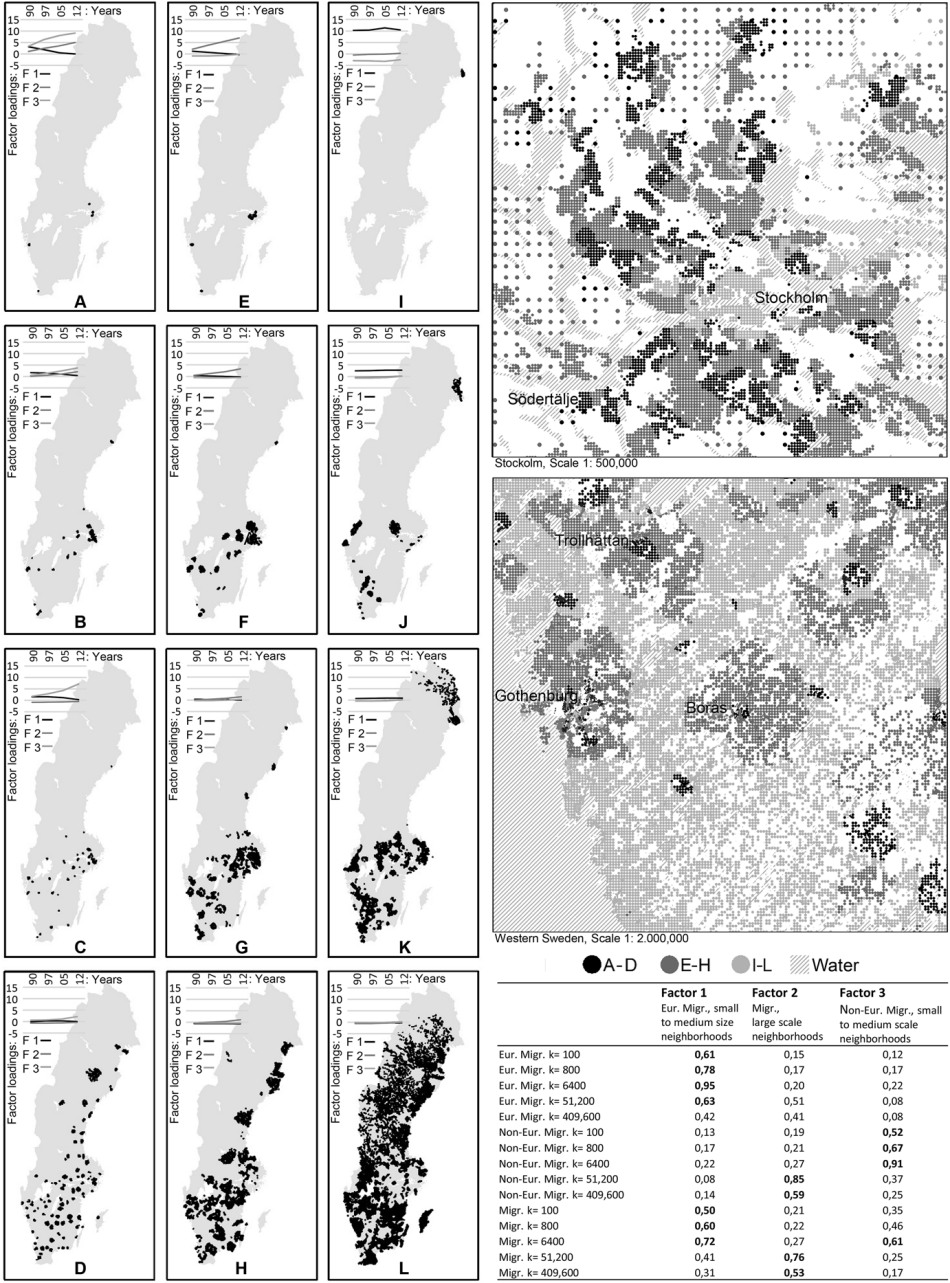
Classification of locations based on neighbourhood composition 1990–2012. *Note* Maps A–D show clusters with high scores on factor 3; non-European migrants (F 3). Maps E–H show clusters with high scores on factor 2; all migrants, large-scale neighbourhoods (F 2). Maps I–K show clusters with high scores on factor 1; European migrants, large-scale neighbourhoods (F 1). Map L shows clusters with low scores on all factors, i.e. few migrants. Average factors scores are shown in the inset diagrams. The maps on the right side show the cluster composition in Stockholm and south western Sweden. The table shows factor loadings. For a version in colour see Nielsen (2018). *Source data*: Swedish register data, authors’ calculations

Figure 5 presents the results of the classification of populated grids cells based on the composition of the neighbourhood population in 1990, 1997, 2005, and 2012 across different geographical scales. This classification groups together locations that over time follow similar trajectories in the composition of their neighbourhood population. The 12 different types have been labelled with the letters A to L. These different types are described in more detail below. The classification can also be examined by looking at the small inset diagrams in each map which show the mean factor scores for the three factors used in the classification. The loadings for these factors are displayed in the bottom right corner of Fig. 5.

The inset diagrams in map A to D (first column) show that these clusters are characterised by increasing values on factor 3 (F 3) over time, and this corresponds to increasing proportions of non-European migrants at local neighbourhood scales, in particular at *k *= 800 and *k *= 6400. Types E to H are characterised by increasing values for factor 2 (F 2) which corresponds to increasing proportions of all migrants and non-European migrants at the scale of large urban areas, in particular at *k *= 51,200 but also at *k *= 409,600. Finally, types I–L are characterised by low values for factors 2 and 3, but with the exception of type L, which has relatively high scores for factor 1, corresponding to high proportions of European migrants at local neighbourhood scales.

The clusters in the first column (A–D) of Fig. 5 are in descending order with respect to factor 3 (non-European, local neighbourhood), while clusters E–H are in descending order with respect to factor 2 (non-European and all migrants for a large urban area), and clusters I to L are in descending order with respect to factor 1 (European migrants, local).

Maps A–D, presented in the first column, show that the location of clusters characterised by increasing proportions of non-European migrants at the scale of local neighbourhoods are linked to different levels of the urban hierarchy. Clusters of type A, with the highest proportion of non-European migrants, are found only in the four major metropolitan areas: Stockholm, Gothenburg, Malmö and Uppsala. Clusters of type B, with somewhat lower proportions of non-European migrants are found in the major metropolitan areas and a selection of the major cities. Clusters of type C are found in a broader range of the larger cities, whereas clusters of type D, with relatively low but increasing proportions of non-European migrants, are relatively well-represented also in smaller urban centres.

The more detailed locations of clusters A–D are also presented in the map for Stockholm and the map for western Sweden in the right-most panel of Fig. 5. As shown in the legend, clusters A to D are represented by the darkest shade. These two maps again show that clusters with increasing proportions of non-Europeans are found in the urban areas of Sweden, with western and South Stockholm having the largest concentrations of clusters having high proportions of non-European migrants at the scale of local neighbourhoods.

The second column of maps presents the locations of clusters that have high and increasing proportions of all migrants and non-European migrants at the scale of large urban areas, but relatively low proportions of these migrant groups at the scale of local neighbourhoods. As can be seen in maps E to H, the locations of these clusters are closely associated with the locations of clusters A to D. Thus, clusters of type E are found close to clusters of type A; clusters of type F are found close to clusters of type B; and so on. This is not surprising since having a high proportion of migrants at the scale of urban areas is only possible if concentrations of migrants exist nearby.

The third column of maps in Fig. 5 presents the locations of clusters that have low proportions of non-European migrants both at the scale of local neighbourhoods and at the scale of urban areas, with maps I, J, and K showing clusters that have relatively high proportions of European migrants. As shown in maps I to K, clusters with high proportions of European migrants are mainly found in two regions: first, in regions bordering or close to Finland, Norway, and Denmark, and, second, in traditional manufacturing regions in Sweden. Thus, the locations of these clusters reflect cross-border migration and the substantial flows of European labour migrants to Sweden in the post-World War II period. Finally, map L shows locations that have low migrant proportions overall. These are locations that have been relatively untouched by international migration flows during the last three decades and they are mostly found in locations at some distance from the major metropolitan areas, and at long distances from the border regions.

In 2012, only about 20% of the Swedish population lived in neighbourhoods with a population largely unaffected by international migration (cluster L). About 15% of the population lived in locations mostly affected by European migration (clusters I, J, and K), and close to 30% lived in locations that had experienced increasing proportions of non-European migrants in their local neighbourhood (clusters A to D), while about 35% lived in locations with low proportions of non-Europeans in their local neighbourhood but with increasing proportions of non-European migrants at neighbourhood scales that correspond to the size of urban areas (clusters E to H). These figures demonstrate that the arrival of migrants has changed almost the whole country from being relatively homogenous into one where most inhabitants live in neighbourhoods with important country-of-birth diversity, locally or at the scale of urban areas.

In the different clusters presented in the maps in Fig. 5, four different patterns can be singled out. First, in 2012, about 16% of the population in Sweden lived in areas with a stable presence of migrants coming from different European countries, clusters I, J, and K. These areas are found mainly in regions that had high rates of immigration in the 1950s, 1960s, and 1970s, often dominated by flows of labour migration directed to the Swedish manufacturing industry. As evidenced by the declining dissimilarity indices, European migrants fit into the theory of spatial assimilation quite well. That is, European migrants live in neighbourhoods that are becoming increasingly similar to that of the general population over time. This group’s longer stay in Sweden, in combination with adaptive residential mobility (Turner and Hedman 2014), and less discrimination in society compared to non-European migrants, may add to the explanation of their residential mobility patterns.

Second, some parts of Sweden have been relatively unaffected by international migration, cluster L. Such areas are generally found in the Swedish countryside in locations that are far from population agglomerations. In 2012, these areas constituted 41% of the populated grid cells, while their population proportion was only 22%.

Third, many locations in the major metropolitan areas but also in large to medium-sized cities, and in some smaller towns experienced a significant increase in the number of non-European migrants among the closest 100 to 6400 neighbours, clusters A–D. In 2012, 25% of the population in Sweden lived in locations belonging to these clusters. The dissimilarity indices show that in comparison to European migrants, non-European migrants are more unevenly distributed. Therefore, as the group of non-Europeans has expanded, an increasing proportion of them now live in neighbourhoods with very high concentrations of other non-European migrants. This has happened even though the distribution of the non-European migrant population has become more even, as measured by the dissimilarity index. Such patterns may indicate that mechanisms such as discrimination and stigmatisation are at play for some of these migrants, as outlined in the place stratification theory.

Fourth, in 2012, 37% of the population in Sweden lived in areas that had experienced little change in the proportion of non-European migrants among their closest neighbours but significant increases in the proportion of non-European migrants among their closest 51,200–409,600 neighbours, clusters E–H. That is, more than a third of the population lived in small-scale neighbourhoods dominated by Swedish-born residents, but in large-scale geographical contexts that were diverse. These areas are found in the major metropolitan areas, but also in and close to small, medium-sized cities, and in large cities.

## Concluding Discussion

At times when migration is high, a recurrent question is how cohesion, integration and inclusion take place in affected societies. These questions are now on the agenda in Sweden due to the annual entry of about 100,000 immigrants, with an increasing proportion of the migrant population originating from outside of Europe. In this context, the aim of this paper was to analyse how a migrant population that is both expanding and changing in composition has affected the composition of Swedish neighbourhoods in different parts of the country and at different scales. Neighbourhoods, in this paper, have been constructed using the Equipop approach which implies that they vary in size with population density. The analytical tools used included percentile plots, the dissimilarity index and maps.

Our main finding is that although the increasing size of the total migrant and non-European migrant population has produced an increasing number of neighbourhoods with very high concentrations of migrants across different neighbourhood scales, often located in metropolitan areas, changes in segregation based on the dissimilarity index point to a dominant trend of declining segregation. The reason for this is that an increasing local presence of non-European migrants is not a phenomenon restricted to immigrant-dense neighbourhoods, but has affected a large majority of Swedish neighbourhoods, many of them located in small to medium-sized urban agglomerations and in larger cities. Our analysis also reveals that for a large part of the Swedish population, the expansion of the migrant population has had stronger effects on the presence of foreign-born at larger neighbourhood scales than on smaller neighbourhood scales.

One important result based on the percentile plots is that there are increasing differences across neighbourhoods in the proportion of migrants and non-European migrants specifically. Changes in the dissimilarity index, on the other hand, point to declining segregation trends in Sweden that are comparable to trends in other countries and to earlier Swedish studies (see Sect. 2). So, *while the unevenness (measured by the dissimilarity index) is decreasing, differences in neighbourhood characteristics are increasing,* see Fig. 4. Consequently, although the segregation trend based on the dissimilarity index has been towards a more even representation of the non-European migrant population across neighbourhoods, the expanding non-European migrant population has led to *increasing* gaps in the concentration of migrants in different neighbourhoods. These diverging results—decreasing segregation measured as unevenness (dissimilarity index) and increasing segregation when it comes to differences in concentration between areas—point to the need for a discussion of how to assess segregation trends. In this paper we measured segregation in a different way compared to most previous Swedish segregation studies, employing a multi-scalar approach (Chaix et al. 2009; Clark et al. 2015; Fowler 2016) and using individualised neighbourhoods; thereby giving a comprehensive view of segregation trends, and providing a basis for a discussion of how migration has influenced migrant segregation trends in Sweden.

As we have argued in Sect. 4.4, seeing segregation as a variation in the concentration of foreign-born can be just as important as trends in the dissimilarity index based on an unevenness definition of segregation. For example, concentration levels in migrant-dense neighbourhoods are higher at small-scale levels, for instance when looking at the 100 closest neighbours, for all migrant populations, and small-scale segregation is especially high for non-European migrants. This will have implications for the possibility of contact between migrants and natives, and leads to stigmatisation of neighbourhoods. At the same time, the *decrease* in segregation has been strongest for small-scale neighbourhoods for non-Europeans, as shown by the dissimilarity index.

From a policy point of view, there is interest in segregation because high levels of segregation imply reduced contact between different populations, and this could have negative effects on social cohesion, democracy and equality in a society. Another, potentially negative effect of segregation could be that reduced contact between populations can make it more difficult for some individuals to take advantage of the positive opportunities that are associated with being socially and economically integrated into a society.

In contrast to most previous studies of segregation in Sweden, our analysis has not looked only at metropolitan areas, but included rural areas as well. We see this as an advantage since processes of segregation are not restricted to residential mobility within urban regions but also include interregional migration. Indeed, as our analysis demonstrates, if non-metropolitan areas had been excluded, many of the most segregated areas would not have been identified. In addition, we would argue that the broader scope of our analysis has been to shed new light on the specificity of metropolitan area segregation. That is, it is not only in metropolitan areas that one finds areas with very high migrant concentrations. What characterises metropolitan areas is the combination of locations with relatively low migrant concentrations at smaller neighbourhood scales with relatively high concentrations of migrants at larger neighbourhood scales. This implies that non-migrants living in a metropolitan area will experience a greater variation in ethnic composition across neighbourhood scales than many non-migrants outside these regions do.

In relation to these concerns, we would argue that residential segregation measures based on individualised neighbourhoods have an advantage. Previous research has shown that the characteristics of individual neighbourhoods are of importance for an individual’s opportunities concerning matters such as work and education (Wimark et al. 2017). The risk of neighbourhood effects being substantially different is higher when areas in which people live are worlds apart as regards population composition (Andersson and Malmberg 2018). This, we claim, is an important argument for not using only DI to measure segregation of migrants over time.

Thus, the individualised neighbourhood approach is helpful in highlighting the fact that large migration flows influence the neighbourhood composition on different geographical scales and in different locations. This contributes to variation in how the geographical context of different individuals is changing. Whereas some individuals experience change in their close neighbourhood, others experience little change nearby but more substantial change at larger geographical scales. Clearly, this diversity of experiences is important to consider when the effects of large migration flows on host societies are analysed.

## Electronic supplementary material

Below is the link to the electronic supplementary material.

Supplementary material 1 (DOCX 16 kb)

## Footnotes

1 A more elaborate note on the interpretation of the dissimilarity index computed when using individualized neighbourhoods compared to administrative units is available as online supplementary material.
