# Supplementary material for: Residential Segregation of European and Non-European Migrants in Sweden: 1990–2012
Source: Eur J Popul. 2018 Mar 21;34(2):169–93. doi: 10.1007/s10680-018-9478-0 (PMC5932106; doi:10.1007/s10680-018-9478-0)
Supplement: Supplementary file 1 — Supplementary material 1 (DOCX 16 kb) [file 10680_2018_9478_MOESM1_ESM.docx]

Malmberg et al.

# Residential Segregation of European and Non-European Migrants in Sweden 1990-2012

# Note on the interpretation of the dissimilarity index when computed using data for individualized neighborhoods with equal population size

The dissimilarity index (DI) is the most widely used measure of residential segregation (Massey & Denton, 1988). It is a measure that compares the distribution of two distinct groups across a number of neighborhoods. In order to compute the DI one needs data on the population composition of residential neighborhoods, for example in terms of different racial groups. When one has such data the dissimilarity index can be computed in four steps. In the first step one computes the total number individuals belonging to two different groups, for example black and white. The second step is to compute what percentage of the total black and the total white population reside in each neighborhood. In the third step one computes the absolute difference between the neighborhood percentage of the total black population and the neighborhood percentage of the total white population. In the fourth and final step one takes the sum of these absolute differences and divides the sum by 2 to obtain the dissimilarity index.

A popular interpretation of the dissimilarity index is that is states what proportion of, for example, the black or white population, needs to be relocated in order to achieve a situation where the percentage of the total black population living in a neighborhood is the same as the percentage of the total white population that lives in the neighborhood. That this is the case can be understood if one considers that by, for example, relocating whites from neighborhoods where they are overrepresented and moving them to neighborhoods where they are underrepresented, will suffice for establishing an even distribution of blacks and whites across neighborhoods.

It should be noted, however, that this popular interpretation is actually a mathematical property of the dissimilarity index. It does not constitute a practical scheme for establishing an even distribution. The reason is that if, in theory, individuals are removed from neighborhoods where they are overrepresented this will amount to reducing the population in those neighborhoods, often drastically. Consider for example a neighborhood with only white residents. This neighborhood’s share of the total black population will be zero, and thus to eliminate white overrepresentation the white population in this neighborhood needs to be reduced to zero too. That is, there needs to be a total depopulation.

Traditionally, the dissimilarity index is computed using population figures for fixed geographical subdivisions such as census tracts. However, because of

the modifiable areal unit problem (MAUP) there is a risk that statistics based on aggregates for fixed geographical subdivisions, including the dissimilarity index, are more dependent on how the boundaries of those areas have been constructed than on the underlying geographical distribution. Consider, for example the chess board. If single squares are considered as geographical entities, a chess board is an example of perfect segregation. If instead two by two squares are used to measure segregation, the chess board gives an example of perfect integration. In order to circumvent the modifiable areal unit problem, one can instead use individualized scalable neighborhoods as a starting point for segregation measurement. That is, neighborhoods are constructed as buffers around individual locations and are expanded until they contain a pre-defined number of nearest neighbors. Different statistics can then be computed for the buffer population. To construct individualized neighborhoods one needs detailed geocoded data. The main advantage of this method is that one enables the construction of neighborhoods in the same way in different contexts and, thus, enables the provision of standardized measures of segregation. The dissimilarity index is one measure that can be computed from data on the population composition of individualized neighborhoods. This can be done by considering every individualized neighborhood as a separate area. For example, consider the case when there are two racial groups, black and white, and where each individualized neighborhood contains the 200 nearest neighbors. Here, the first step is to compute the number of blacks and whites in each individualized neighborhood based on the proportion of blacks and whites found in the buffer population. Second, the sums of blacks and the sum of whites across all individualized neighborhoods is computed and these sums are used to compute each individualized neighborhood share of the aggregate black and the aggregate white population. Then the dissimilarity index can be computed by taking the absolute difference between the share of the aggregate black and the share of the aggregate white population and summing across all neighborhoods. Note that in this computation, the aggregate black and the aggregate white population obtained by summing the number of black and white in each neighborhood will not be the same as the total black and white population of the geographical unit under study. However, the dissimilarity index will still provide a measure of the extent to which blacks and whites are distributed similarly across neighborhoods.

Massey, D. S., & Denton, N. A. (1988). The dimensions of residential segregation. *Social Forces, 67*(2), 281-315.
